# Prevalence and genetic diversity of *Pentatrichomonas hominis* in pig populations in Guangdong and Anhui Provinces, China

**DOI:** 10.1051/parasite/2025027

**Published:** 2025-06-11

**Authors:** Pengyun Lu, Yibin Zhu, Haiming Cai, Hanqin Shen, Siyun Fang, Dingai Wang, Zhuanqiang Yan, Shenquan Liao, Nanshan Qi, Minna Lv, Xuhui Lin, Yongle Song, Xiangjie Chen, Jianfei Zhang, Juan Li, Mingfei Sun

**Affiliations:** 1 Key Laboratory of Livestock Disease Prevention of Guangdong Province, Key Laboratory of Avian Influenza and Other Major Poultry Diseases Prevention and Control, Ministry of Agriculture and Rural Affairs, Institute of Animal Health, Guangdong Academy of Agricultural Sciences 510640 Guangzhou China; 2 Guangdong Guangken Animal Husbandry Group Co., Ltd. 510000 Guangdong China.; 3 Wen’s Group Academy, Wen’s Foodstuffs Group Co., Ltd., Xinxing 527400 Guangdong China.

**Keywords:** *Pentatrichomonas hominis*, Pig infection, Zoonotic potential, Prevalence, Genetic diversity

## Abstract

*Pentatrichomonas hominis* is a protozoan parasite that infects the gastrointestinal tract of humans and mammals, causing abdominal pain and diarrhea. However, its presence in pigs and its potential as a pathogen causing diarrhea in piglets have not been well studied. This study aimed to investigate the prevalence of *P. hominis* in pigs and its potential for zoonotic transmission. A total of 406 pig fecal samples were collected from four pig farms located in Guangdong and Anhui Provinces. Fecal DNA extraction was carried out using a commercially available kit. A nested PCR methodology was employed to detect the presence of *P. hominis* infection. Samples that tested positive were subsequently subjected to sequencing, and the genetic characteristics of the 18S ribosomal RNA (rRNA) gene were analyzed. The overall infection rate of *P. hominis* was 34.98% (142/406), in Guangdong Province 28.47% (80/281), and in Anhui Province 49.60% (62/125). Among different age groups, suckling piglets had the highest infection rate at 40.24% (68/169). Genetic analysis of the *P. hominis* isolates showed that the PH-1 genotype was predominant and had a high degree of similarity to *P. hominis* sequences obtained from humans, cats, and pigs, indicating the potential for zoonotic transmission. The high infection rate and genetic diversity highlight the need for effective control measures in pig farming to reduce parasite transmission and zoonotic risk.

## Abstract (fr)

*Pentatrichomonas hominis* est un parasite protozoaire qui infecte le tractus gastro-intestinal des humains et des mammifères, provoquant douleurs abdominales et diarrhées. Cependant, sa présence chez les porcs et son potentiel pathogène causant des diarrhées chez les porcelets n’ont pas été bien étudiés. Cette étude visait à évaluer la prévalence de *P. hominis* chez les porcs et son potentiel de transmission zoonotique. Au total, 406 échantillons fécaux de porcs ont été collectés dans quatre élevages porcins des provinces du Guangdong et de l’Anhui. L’extraction de l’ADN fécal a été réalisée à l’aide d’un kit disponible dans le commerce. La méthode de PCR nichée a été utilisée pour détecter la présence d’une infection à *P. hominis*. Les échantillons positifs ont ensuite été séquencés et les caractéristiques génétiques du gène de l’ARN ribosomique (ARNr) 18S ont été analysées. Le taux d’infection global de *P. hominis* était de 34,98 % (142/406), avec 28,47 % (80/281) dans la province du Guangdong et 49,60 % (62/125) dans la province de l’Anhui. Parmi les différents groupes d’âge, les porcelets allaités présentaient le taux d’infection le plus élevé, soit 40,24 % (68/169). L’analyse génétique des isolats de *P. hominis* a montré que le génotype PH-1 était prédominant et présentait un degré élevé de similitude avec les séquences de *P. hominis* obtenues chez l’homme, le chat et le porc, indiquant un potentiel de transmission zoonotique. Le taux d’infection élevé et la diversité génétique soulignent la nécessité de mesures de contrôle efficaces dans les élevages porcins pour réduire la transmission des parasites et le risque zoonotique.

## Introduction

Trichomonads, a group of protozoans, are commonly found as symbionts or parasites inhabiting the warm, moist, anaerobic environments of the digestive or genitourinary tracts of humans and animals [16]. These microorganisms have been extensively studied in the field of veterinary medicine. Trichomonads possess hydrogenosomes in place of mitochondria and can have up to six flagella [21]. Hydrogenosomes are specialized anaerobic organelles that play a critical role in energy production, fundamentally distinguishing trichomonads from mitochondria-bearing eukaryotes. The multiple flagella characteristic of these organisms facilitate their motility and enhance their capacity for host colonization. Among trichomonads, *P. hominis* is a pathogenic species that induces gastrointestinal symptoms in both humans [1, 25] and animals [2]. Recent advances in molecular detection techniques and increased awareness of the zoonotic potential of this parasite have led to growing interest in *P. hominis* research.

*Pentatrichomonas hominis* primarily colonizes the large intestines of mammals and is mainly transmitted through the fecal-oral route [10]. Despite its initial classification as a commensal protozoan, it has been found to cause diarrhea and gastrointestinal or pulmonary diseases in humans [19]. Approximately 41.54% of *P. hominis* infections have been documented in Chinese patients with gastrointestinal cancer [25]. Additionally, *P. hominis* has been associated with irritable bowel syndrome [20], systemic lupus erythematosus [11], and rheumatoid arthritis [3] in humans. The zoonotic and pathogenic potential of *P. hominis* has prompted an increase in studies investigating its prevalence and pathogenicity across different vertebrates. This parasite has been identified in dogs [19], cats [2], cattle [14], pigs [21], monkeys, as well as various other animals, including goats [19], tigers [24], and farmed wildlife [17]. Although the pathogenicity of *P. hominis* in animals remains poorly understood, it has been suggested to contribute to enteric diseases in pigs [12, 15]. Given the significant role of pigs in the global food production industry, understanding the distribution and epidemiology of *P. hominis* infections in pigs is crucial for disease prevention and control strategies. Additionally, studying its prevalence in pigs is essential for evaluating the risk of transmission to humans.

This study aimed to investigate the infection and prevalence of *P. hominis* in pig populations within the Guangdong and Anhui Provinces. Stool samples obtained from the pigs were examined using nested polymerase chain reaction (PCR) techniques, targeting specific genes (partial 18S rRNA of *P. hominis*) for the precise identification of *P. hominis* infections. The findings of this study offer valuable insights for the development of effective measures aimed at preventing and controlling this parasite in pigs. Notably, this study represents one of the pioneering initiatives focused on investigating *P. hominis* infection and prevalence, particularly in pigs within the Guangdong and Anhui Provinces of China. Furthermore, it provides additional evidence regarding parasitic infections in this particular livestock species.

## Materials and methods

### Ethics

The collection of fecal samples from pigs in this study did not require specific approval from an animal ethics committee according to local regulations, as samples were collected during routine diagnostic procedures by veterinarians after obtaining permission from the farm owner. No additional procedures were performed on animals for research purposes, and sample collection did not cause any distress beyond standard veterinary care. This study was conducted in accordance with the guidelines of the Institute of Animal Health, Guangdong Academy of Agricultural Sciences, and complied with all relevant local legislation and institutional requirements. All subsequent experiments were performed on fecal DNA in laboratory settings and did not involve any direct animal experimentation.

### Collection of fecal samples

A total of 406 pig fecal samples were collected from four pig farms located in Guangdong and Anhui Provinces. These comprised 84 samples procured from a pig farm in Zhaoqing, Guangdong, 70 samples obtained from a pig farm in Jiangmen, Guangdong, 127 samples sourced from a pig farm in Yunfu, Guangdong, and 125 samples from a pig farm in Lu’an, Anhui. All farms were situated at least 10 km from residential areas to minimize direct human contact. The selected farms were comprehensive breeding operations housing pigs of all age groups. The sampling strategy employed a random approach that included both healthy and diarrheic animals to ensure representative population coverage. The fecal samples were collected using aseptic techniques and subsequently stored at a temperature of −20 °C until further analysis.

### Extraction of fecal DNA

To extract fecal DNA, 0.2 g of each fecal sample was weighed and thoroughly mixed before being placed in a 1.5 mL centrifuge tube. A MagaBio Fecal Pathogen DNA/RNA Purification Kit (Hangzhou Borui Technology Co., Ltd., Hangzhou, China) was used for the extraction process. This process involved the addition of an equal volume of glass beads, PK, and lysis buffer to the centrifuge tube. The fecal sample was then lysed by shaking at 60.0 Hz for 3 min, followed by incubation at 65 °C for 10 min. After the addition of a DA Buffer and a 5-min incubation on ice, the supernatant was separated via centrifugation and then transferred to a 96-well plate. The automated nucleic acid extraction program was executed using an NPA-32P nucleic acid extraction and purification system, which involved several steps, such as magnetic bead separation and washing. The elution liquid was collected from the 5th and 11th columns and stored at −20 °C for subsequent use. These steps ensured the efficient extraction of fecal DNA and the preservation of its integrity for downstream analysis. The use of a MagaBio fecal pathogen DNA/RNA purification kit, NPA-32P nucleic acid extraction, and fully automated nucleic acid extraction and purification system (Hangzhou Borui Technology Co., Ltd.) was complemented by the implementation of standardized procedures.

### Detection of fecal genomic DNA samples using nested PCR

To identify the presence of *P. hominis*, the extracted DNA was used as a template, and the identification method described by Li *et al.* [12, 15] was followed. The primary primers, FF (5′–GCGCCTGAGAGATAGCGACTA–3′) and RR (5’-GGACCTGTTATTGCTACCCTCTTC–3′) were synthesized by Guangzhou IGE Biotechnology Co., Ltd. for the nested PCR process, the secondary primers were HF (5′–TGTAAACGATGCCGACAGAG–3′) and HR (5′–CAACACTGAAGCCAATGCGAGG–3′) (Table 1). The PCR reaction was conducted using rTaq mix (TaKaRa, Kyoto, Japan). The initial round of PCR was conducted under the following conditions: a pre-denaturation phase at 95 °C for 10 min, followed by cycling conditions of 95 °C for 1 min, 60 °C for 1 min, and 72 °C for 1 min, with a total of 30 cycles. The process culminated in an extension step at 72 °C for 10 min. Subsequently, the second round of PCR amplification was conducted using the initial round of PCR products as a template. The conditions for the second round of PCR were as follows: pre-denaturation at 95 °C for 10 min, followed by cycling conditions of 95 °C for 30 s, 55 °C for 30 s, and 72 °C for 30 s, for a total of 30 cycles. The reaction was concluded with an extension step at 72 °C for 10 min. The PCR reaction system consisted of 12.5 μL of Premix rTaq, 1 μL of FF/HF (10 μM), 1 μL of RR/HR (10 μM), 9.5 μL of ddH_2_O, and 1 μL of the first round of PCR amplification products diluted 1:10 in nuclease-free water as template, resulting in a total volume of 25 μL.

**Table 1:** Primers used for nested PCR in this study.

Primer	Sequence (5’→3’)	Gene	Product size	Reference
Primary	FF: GCGCCTGAGAGATAGCGACTA	18S rRNA	919 bp	Li *et al.* [15, 18]
	RR: GGACCTGTTATTGCTACCCTCTTC			
Secondary	HF: TGTAAACGATGCCGACAGAG	18S rRNA	339 bp	Li *et al.* [15, 18]
	HR: CAACACTGAAGCCAATGCGAGG			

### Detection of PCR products

A sufficient amount of 50× TAE buffer was diluted to 1× TAE buffer and used to prepare a 2% agarose gel. Once the gel solidified, 10 μL of the PCR product were added to the gel wells. A 500-bp DNA ladder (TaKaRa, Dalian, China) was used as a molecular weight standard. Electrophoresis was performed at a constant voltage of 120 V for approximately 30 min. The gel was stained with GelStain Blue (TransGen, Beijing, China) at 1:10,000 dilution. Following electrophoresis, the gel was examined and photographed using a Tanon 2500 BR Multifunctional gel imaging analyzer (Tanon, Shanghai, China) equipped with UV light. For PCR quality control, we included positive controls (plasmid containing the cloned 18S rRNA gene fragment of *P. hominis* verified by sequencing at 10 ng/μL) and negative controls (nuclease-free water). Each PCR run included these controls in triplicate to ensure reliability and reproducibility of results.

### Sequencing and sequence analysis

Positive samples were purified using a HiPure Gel Pure DNA Mini Kit (Magen, Guangzhou, China). The resulting purified products were sent to Guangzhou IGE Biotechnology Co., Ltd. for sequencing. The sequencing results were assembled and aligned using DNAMAN 6.0 sequence analysis software (Lynnon Biosoft, San Ramon, CA, USA).

### Genetic evolutionary analysis

MEGA-X software (Pennsylvania State University, University Park, PA, USA) was used to align the 18S ribosomal RNA (rRNA) gene sequences with reference sequences. The neighbor-joining method was employed to analyze the evolutionary relationships between the sequences. The stability of the phylogenetic tree was tested using 1,000 bootstrap replicates and the best-fit model. The genetic distance between sequences was calculated using the Kimura-2-parameter nucleotide substitution model.

### Statistical analysis

Statistical analysis was performed using the chi-square test in IBM SPSS Statistics 26.0 software (IBM Corp., Armonk, NY, USA) to compare the infection rates among different groups. A *p*-value of less than 0.05 was considered statistically significant.

## Results

### Analysis of the investigation results of *P. hominis* infection in pigs

**Figure 1:**
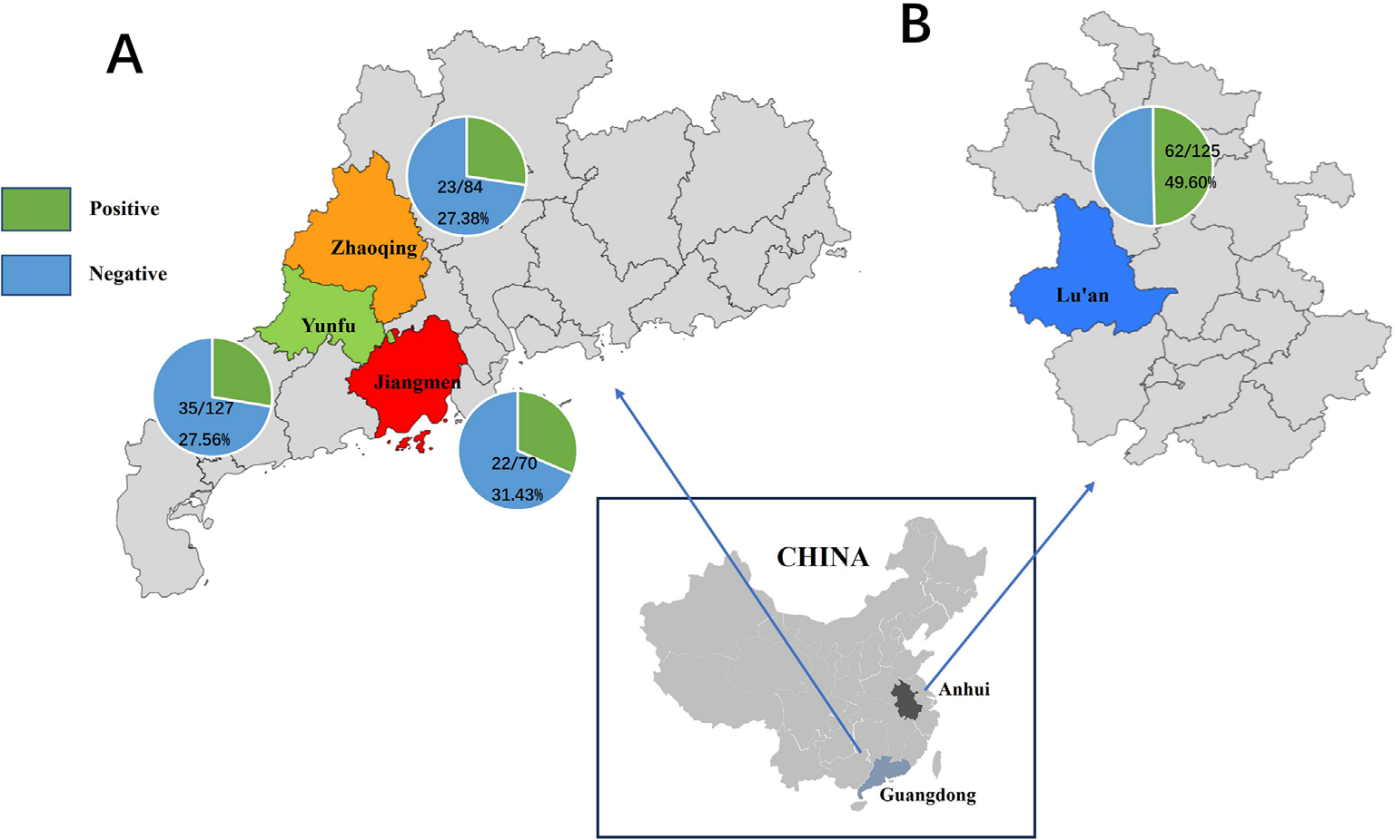
Distribution of *Pentatrichomonas hominis* infection in pigs across different regions in China. (A) Infection rate of *P. hominis* in selected areas of Guangdong Province. (B) Infection rate of *P. hominis* in selected areas of Anhui Province. The infection rate was determined through PCR analysis of pig fecal samples using specific primers for the 18S rRNA gene of *P. hominis*. The number of samples tested and the percentage of positive samples are indicated for each region.

Nested PCR amplification of the target gene was performed on 406 pig fecal genomic DNA samples, and the results of gel electrophoresis displayed distinct target bands at approximately 339 bp for *P. hominis* positive samples (Figure S1). Table 2 illustrates the infection status of *P. hominis* in pig fecal samples. Among the 406 samples tested, 142 were identified as positive for *P. hominis*, yielding an overall infection rate of 34.98%. The infection rates in various regions were as follows: 27.38% (23/84) in Zhaoqing, Guangdong Province; 31.43% (22/70) in Jiangmen, Guangdong Province; 27.56% (35/127) in Yunfu, Guangdong Province; and 49.60% (62/125) in Lu’an, Anhui Province. A graphical representation of the regional differences in *P. hominis* infection rates is presented in Figure 1. Notably, no significant differences in *P. hominis* infection rates were observed among pigs from different regions within Guangdong Province (X^2^ = 0.402, df = 2, *p* = 0.818). Conversely, the infection rate in Lu’an, Anhui Province was significantly higher than that observed in Guangdong Province, with a significant difference in *P. hominis* infection rates between Guangdong and Anhui Provinces (X^2^ = 17.344, df = 3, *p* = 0.001).

**Table 2:** Detection results of molecular epidemiologic investigation for *P. hominis* in pigs.

Independent Variable	Category	Total Samples	Positive Samples	Negative Samples	Infection Rate (%)	95% Confidence Interval
Region	Zhaoqing	84	23	61	27.38%	18.48–38.37
	Jiangmen	70	22	48	31.43%	21.15–43.77
	Yunfu	127	35	92	27.56%	20.19–36.32
	Lu’an	125	62	63	49.60%	40.59–58.63
Age	Suckling Piglets	169	68	101	40.24%	32.86–48.07
	Nursery Pigs	89	32	57	35.96%	26.26–46.89
	Fattening Pigs	95	30	65	31.58%	22.64–42.03
	Pregnant Sows	53	12	41	22.64%	12.72–36.55
Total		406	142	264	34.98%	30.38–39.87

The infection rate of suckling piglets was 40.24% (68/169), the infection rate of nursery pigs was 35.96% (32/89), the infection rate of fattening pigs was 31.58% (30/95), and the infection rate of pregnant sows was 22.64% (12/53). Notably, suckling piglets exhibited the highest infection rate, while pregnant sows displayed the lowest. However, no significant differences in *P. hominis* infection rates were observed among different age groups of pigs (X^2^ = 6.122, df = 3, *p* = 0.106).

Further analysis of the infection rates among different age groups revealed a significant discrepancy in the infection rates of *P. hominis* among suckling piglets, nursery pigs, fattening pigs, and pregnant sows (X^2^ = 16.706, df = 3, *p* = 0.001). The infection rate was highest in fattening pigs (47.37%) and lowest in pregnant sows (22.64%). These findings suggest that *P. hominis* infection is prevalent in pigs across all age groups, with suckling piglets and fattening pigs experiencing the highest incidence. The lack of a significant difference in infection rates among various age groups suggests that all age groups are equally susceptible to *P. hominis* infection.

### Genetic analysis of *P. hominis* sequences

**Figure 2:**
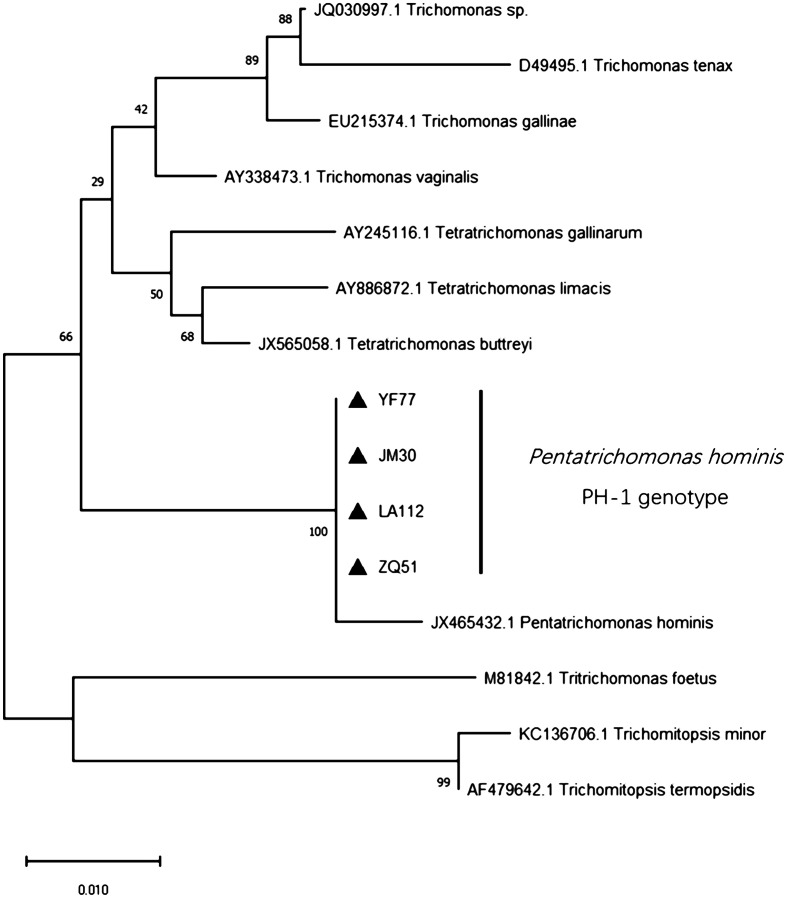
Phylogenetic tree of *Pentatrichomonas hominis* isolates based on the 18S rRNA gene. The reference gene was obtained from GenBank (JX465432). ▲ Genotypes from different regions in this study.

**Figure 3:**
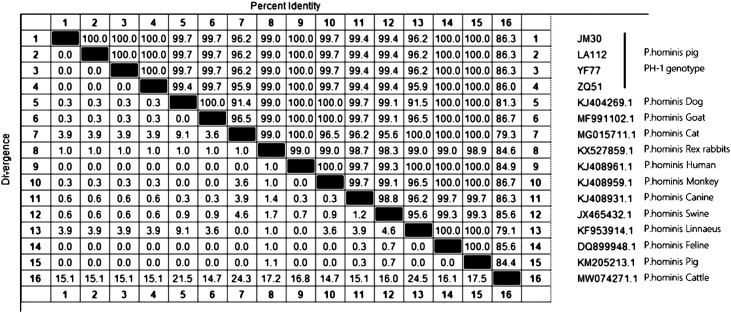
Comparison of the similarity in 18S rRNA gene sequences between pig-derived *Pentatrichomonas hominis* and isolates from other host species.

Out of the 142 positive samples, 141 were successfully sequenced and analyzed. The sequencing results revealed that the predominant genotype was PH-1. Detailed gene sequences are provided in Figure S1. The phylogenetic tree analysis (Fig. 2) revealed a close relationship between the PH-1 genotype and *P. hominis* strains isolated from humans, indicating potential zoonotic transmission. The homology analysis (Fig. 3) showed a high degree of consistency (100%) in the 18S rRNA gene sequences of the PH-1 genotype across various regions when compared to the sequence of *P. hominis* isolated from humans, felines, and pigs (GenBank accession No. KJ408961.1, DQ899948.1, KM205213.1). This high degree of similarity in the gene sequences also indicated the conservation of the genetic material among different *P. hominis* isolates. These findings offer crucial insights into the epidemiology and transmission of *P. hominis* within pig populations, highlighting the need for further investigation of this parasite’s zoonotic potential.

## Discussion

Pig breeding constitutes a significant global industry, with China being the largest producer and consumer of pork. *Pentatrichomonas hominis* infection not only causes substantial economic losses to the pig breeding industry, but also poses risks to human health. Therefore, *P. hominis* infection within pig breeding warrants further attention. *Pentatrichomonas hominis* is a parasitic protozoan with potential to infect humans. It has been documented worldwide, and its presence has been confirmed in various developed and developing regions, including France, Brazil, Egypt, the United States, China, Japan, and South Korea [1, 7, 23]. Although microscopic examination is considered the most direct method for diagnosing parasitic diseases, the large sample sizes and the similar morphology of different trichomonas species in clinical infections make it a challenging approach. With the continuous advancement of molecular biology, PCR detection technology has matured, with the nested PCR detection method proving to be more sensitive and accurate than the optical microscope in identifying trichomonas in feces [5, 18]. In this study, we conducted an epidemiologic survey in Guangdong and Anhui Provinces, China, to investigate the prevalence of *P. hominis* infection in pigs, employing the nested PCR detection method. Our results revealed a high *P. hominis* infection rate in pigs, with significant variations observed among different regions and age groups.

The collection of fecal samples from multiple locations and farms allowed for a diverse representation of pig populations and helped ensure the reliability and validity of the study results. The use of appropriate storage conditions played a key role in maintaining the integrity of the fecal samples, thereby facilitating accurate downstream analysis. Our study identified a total *P. hominis* infection rate in pigs of 34.98% (142/406), which is comparatively lower than rates reported in other hosts, such as monkeys (46.67%), minks (48.33%), silver foxes (43.33%), raccoon dogs (53.33%), and marmosets (66%) [9, 16, 17]. This difference may be attributed to the number of samples and the susceptibility of different hosts to *P. hominis*. Additionally, variations in the habitat environment of different hosts could also contribute to these differences. The infection rate of *P. hominis* in pigs investigated in this study was higher than that in other parts of China, and higher than that in Thailand and the Philippines [7, 13, 15, 19]. This difference may be attributed to variations in sample size, age, seasonality, and ecological environment. The specific temperature and humidity in the southern region may be more conducive to the survival and reproduction of protozoan parasites, such as trichomonas. Moreover, differences in farm scale and feeding conditions may also contribute to the varying infection rates of *P. hominis* [8]. Higher stocking densities may promote environmental pollution, leading to the infection and spread of *P. hominis*. In external environments, trichomonas trophozoites can form pseudocysts, which prolong their survival under unfavorable conditions [22]. The high prevalence of pig infection with *P. hominis* can also be explained by analyzing the age of the host. Infected pigs spanned all age groups (suckling piglets to sows), with the highest rate in suckling piglets (40.24%). Pigs were housed in group pens (10–15 pigs/pen), which likely facilitated fecal-oral transmission. No significant difference was observed between pens, suggesting environmental contamination as a key route. Previous reports have indicated that canine infection with *P. hominis* is predominantly associated with clinical symptoms and age factors, with puppies being at a higher risk [6]. A survey of *P. hominis* infection in pet dogs in East China also revealed a significantly higher infection rate in dogs under 12 months compared to dogs over 12 months [16]. Similarly, children aged 3–5 years were found to have a higher infection rate of *P. hominis* [4]. In our study, weaned piglets accounted for about 2/5 of the total age of pigs, and displayed a higher infection rate of 40.24%. This finding is consistent with the findings reported by Li *et al.* [15]. It is important to note that a limitation exists regarding the uneven age distribution of pigs between provinces, which restricts the ability to make robust comparisons of age-related prevalence across different geographical regions. This imbalance, partly resulting from variations in production cycle management across farms, may influence regional comparative results. While the current analysis provides valuable insights into overall prevalence patterns, the age distribution confounding factor should be considered when interpreting geographical differences. Future research would benefit from implementing standardized age cohort sampling to ensure balanced age group representation across provinces, allowing for more accurate assessment of the interaction between geographical factors and age factors in the epidemiology of *P. hominis*. This type of structured approach could yield more definitive insights into whether the observed regional differences persist when age distribution is more carefully controlled.

Analysis of the 18S rRNA gene in *P. hominis* from various pig populations revealed the predominance of the PH-1 genotype. Phylogenetic analysis demonstrated a high degree of consistency between *P. hominis* gene sequences from various regions and those from humans, felines, and pigs (GenBank accession No. KJ408961.1, DQ899948.1, KM205213.1) (100%). Furthermore, PH-1 exhibited high homology with *P. hominis* sequences from other hosts, such as dogs, goats, rabbits, monkeys, canines, pigs, cats, and cattle (GenBank accession No. KJ404269.1, MF991102.1, MG015711.1, KX527859.1, KJ408959.1, KJ408931.1, JX465432.1, KF953914.1, MW074271.1). These minor genetic differences may be influenced by variations in hosts and surrounding environments. Furthermore, the results affirm that *P. hominis* is not host-specific and possesses the potential for zoonotic transmission. Additionally, our findings confirm that pigs can serve as hosts for *P. hominis* and may act as a source of human infection.

## Conclusion

In conclusion, this study revealed a high prevalence (34.98%) and genetic diversity of *P. hominis* in Guangdong and Anhui pig populations. The PH-1 genotype’s homology with human isolates underscores zoonotic risks. Implementing improved farm hygiene and periodic surveillance is critical to mitigate transmission. Further research is needed to clarify transmission dynamics and clinical impacts.

## Data Availability

The nucleotide sequence generated in the present study has been deposited in GenBank (https://www.ncbi.nlm.nih.gov/) under accession number OR649150. The datasets used and/or analyzed during the current study are available from the corresponding author on reasonable request.

## Supplementary material

The supplementary material for this article can be found at https://www.parasite-journal.org/10.1051/parasite/2025027/olm. *Figure S1*. Gel electrophoresis of PCR products amplified from pig fecal samples. Lane M: DNA ladder marker; Lanes 1-406: PCR products from fecal samples of pigs numbered 1-406. The presence of a band at the expected size confirms the presence of *Pentatrichomonas hominis* in the fecal sample.
