# Supplementary material for: Prevalence and genetic diversity of Pentatrichomonas hominis in pig populations in Guangdong and Anhui Provinces, China
Source: Parasite. 2025 Jun 11;32:33. doi: 10.1051/parasite/2025027 (PMC12158214; doi:10.1051/parasite/2025027)
Supplement: Supplementary file 1 — Figure S1. Gel electrophoresis of PCR products amplified from pig fecal samples. Lane M: DNA ladder marker; Lanes 1-406: PCR products from fecal samples of pigs numbered 1-406. The presence of a band at the expected size confirms the presence of Pentatrichomonas hominis in the fecal sample. [file parasite-32-33-s1.pdf]

Supplementary files:

Prevalence and Genetic Diversity of *Pentatrichomonas hominis* in Pig Populations in Guangdong and Anhui Provinces, China

Pengyun Lu [a, b†], Yibin Zhu [a†], Haiming Cai [a], Hanqin Shen [c], Siyun Fang [c], Dingai Wang [c], Zhuanqiang Yan [c], Shenquan Liao [a], Nanshan Qi [a], Minna Lv [a], Xuhui Lin [a], Yongle Song [a], Xiangjie Chen [a], Jianfei Zhang [a], Juan Li [a*], Mingfei Sun [a*]

[a] Key Laboratory of Livestock Disease Prevention of Guangdong Province, Key Laboratory of Avian Influenza and Other Major Poultry Diseases Prevention and Control, Ministry of Agriculture and Rural Affairs, Institute of Animal Health, Guangdong Academy of Agricultural Sciences, Guangzhou, 510640, China; lupengyun@163.com (P.L.); zhuyibin@gdaas.cn (Y.Z.)

[b] Guangdong Guangken Animal Husbandry Group Co., Ltd., Guangdong 510000, China.

[c] Wen's Group Academy, Wen's Foodstuffs Group Co., Ltd., Xinxing, Guangdong 527400, China.

***** Correspondence: lijuan@gdaas.cn (J.L.); sunmingfei@gdaas.cn (M.S.)

† These authors contributed equally to this work.

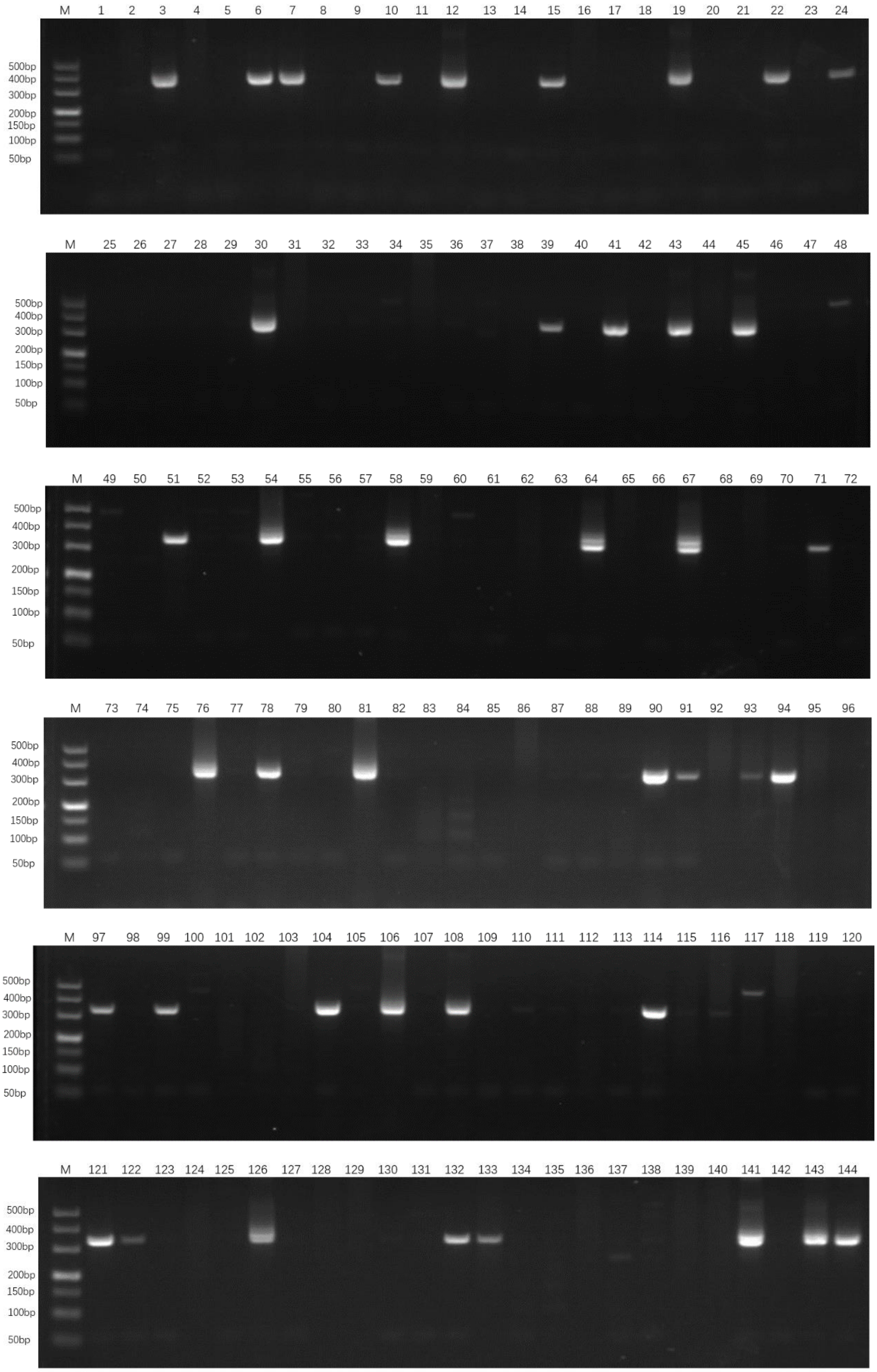
M 1 2 3 4 5 6 7 8 9 10 11 12 13 14 15 16 17 18 19 20 21 22 23 24
500bp 400bp 300bp 200bp 150bp 100bp 50bp
M 25 26 27 28 29 30 31 32 33 34 35 36 37 38 39 40 41 42 43 44 45 46 47 48
500bp 400bp 300bp 200bp 150bp 100bp 50bp
M 49 50 51 52 53 54 55 56 57 58 59 60 61 62 63 64 65 66 67 68 69 70 71 72
500bp 400bp 300bp 200bp 150bp 100bp 50bp
M 73 74 75 76 77 78 79 80 81 82 83 84 85 86 87 88 89 90 91 92 93 94 95 96
500bp 400bp 300bp 200bp 150bp 100bp 50bp
M 97 98 99 100 101 102 103 104 105 106 107 108 109 110 111 112 113 114 115 116 117 118 119 120
500bp 400bp 300bp 200bp 150bp 100bp 50bp
M 121 122 123 124 125 126 127 128 129 130 131 132 133 134 135 136 137 138 139 140 141 142 143 144
500bp 400bp 300bp 200bp 150bp 100bp 50bp

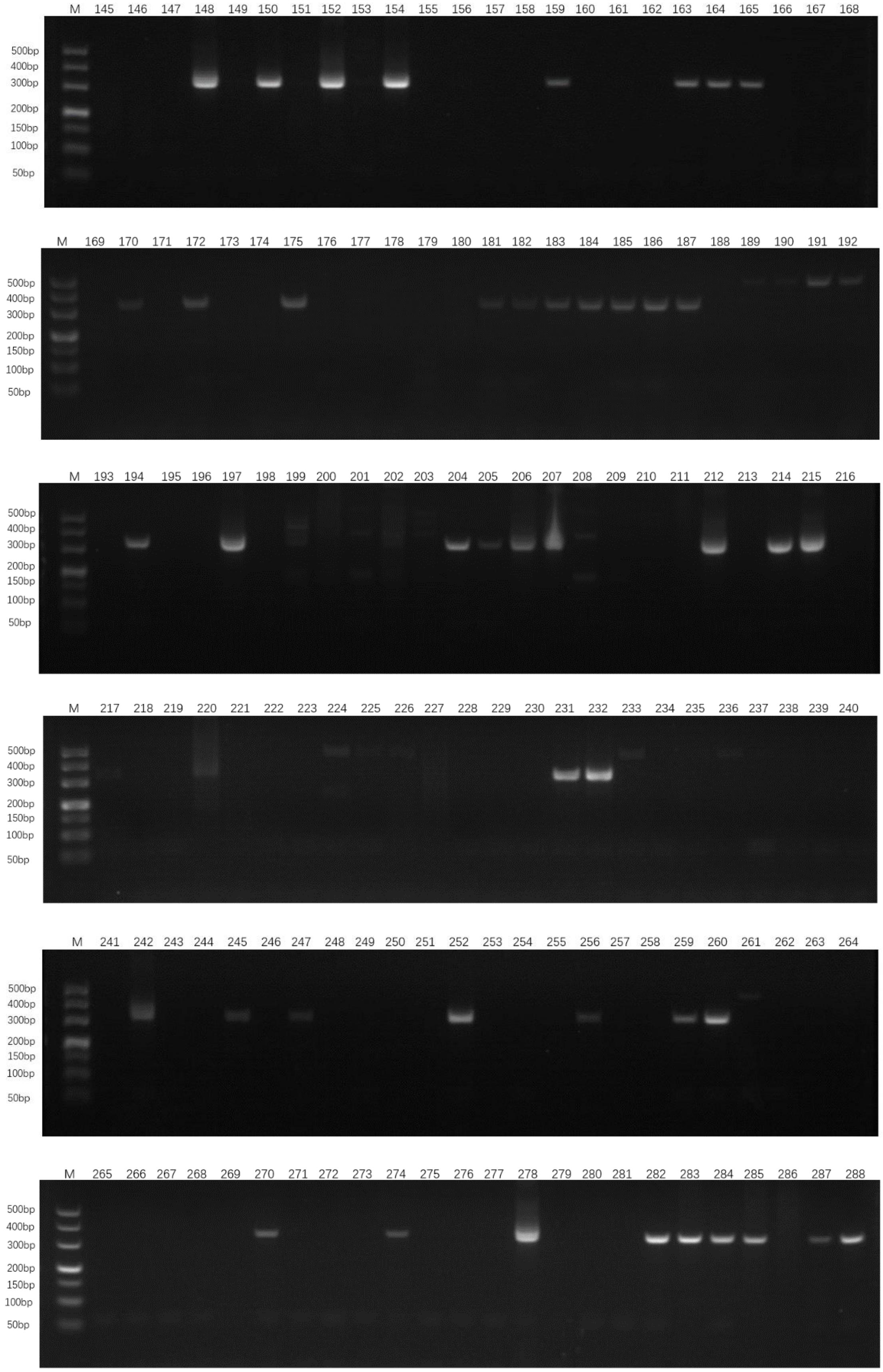
M 145 146 147 148 149 150 151 152 153 154 155 156 157 158 159 160 161 162 163 164 165 166 167 168
500bp
400bp
300bp
200bp
150bp
100bp
50bp
M 169 170 171 172 173 174 175 176 177 178 179 180 181 182 183 184 185 186 187 188 189 190 191 192
500bp
400bp
300bp
200bp
150bp
100bp
50bp
M 193 194 195 196 197 198 199 200 201 202 203 204 205 206 207 208 209 210 211 212 213 214 215 216
500bp
400bp
300bp
200bp
150bp
100bp
50bp
M 217 218 219 220 221 222 223 224 225 226 227 228 229 230 231 232 233 234 235 236 237 238 239 240
500bp
400bp
300bp
200bp
150bp
100bp
50bp
M 241 242 243 244 245 246 247 248 249 250 251 252 253 254 255 256 257 258 259 260 261 262 263 264
500bp
400bp
300bp
200bp
150bp
100bp
50bp
M 265 266 267 268 269 270 271 272 273 274 275 276 277 278 279 280 281 282 283 284 285 286 287 288
500bp
400bp
300bp
200bp
150bp
100bp
50bp

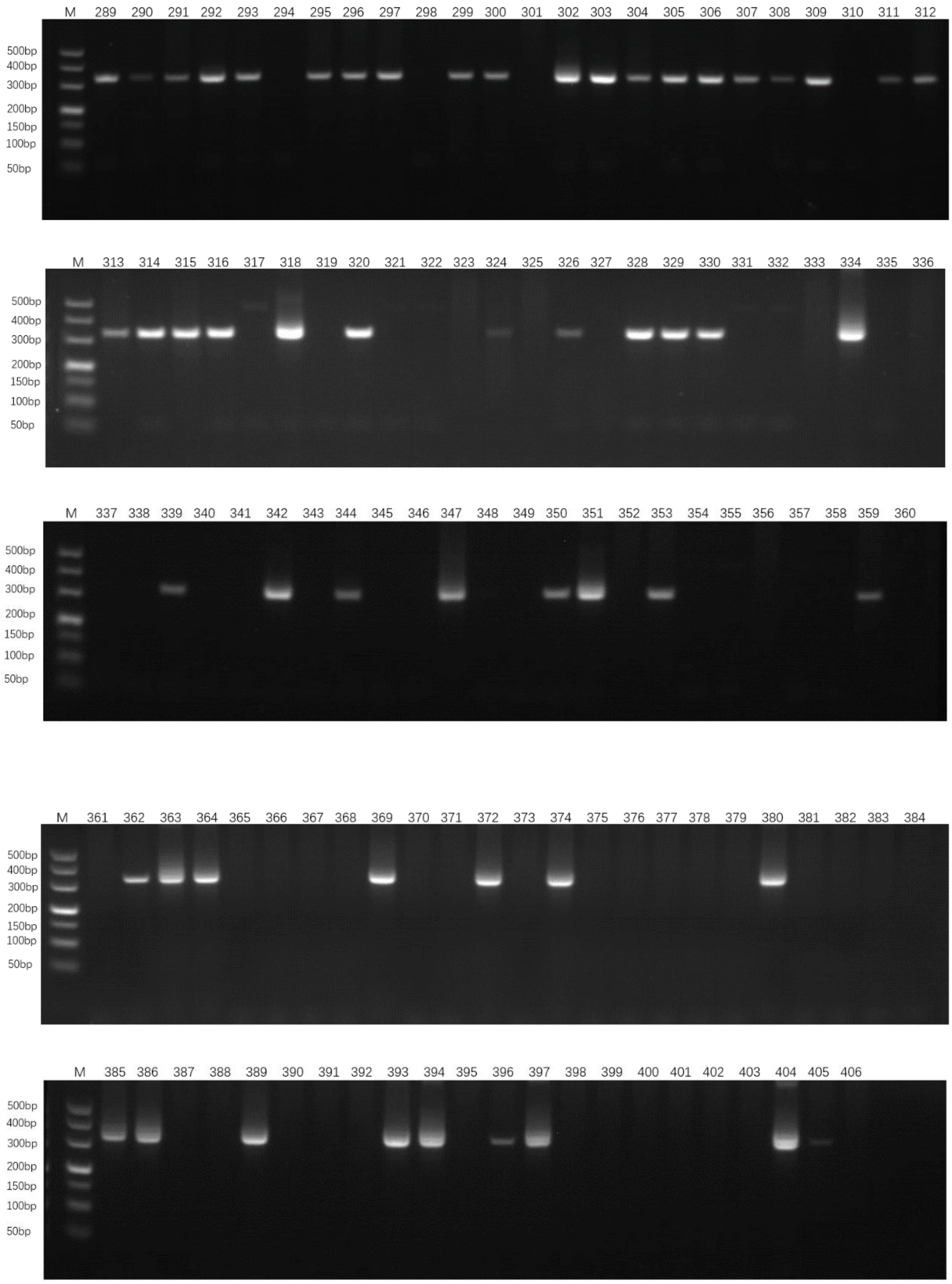

Figure S1. Gel electrophoresis of PCR products amplified from pig fecal samples. Lane M: DNA ladder marker; Lanes 1-406: PCR products from fecal samples of pigs numbered 1-406.The presence of a band at the expected size confirms the presence of *Pentatrichomonas hominis* in the fecal sample.
